# Malignant melanoma arising from a perianal fistula and harbouring a *BRAF *gene mutation: a case report

**DOI:** 10.1186/1471-2407-11-343

**Published:** 2011-08-09

**Authors:** Conrado Martinez-Cadenas, Nuria Bosch, Lucas Peñas, Esther Flores-Couce, Enrique Ochoa, Javier Munárriz, Juan P Aracil, Marcos Tajahuerce, Ramón Royo, Rafael Lozoya, Enrique Boldó

**Affiliations:** 1 Molecular Biopathology Lab, Castellon Province Hospital, Ave. Doctor Clara 19, Castellon, 12002, Spain; 2 Pathology Service, Castellon Province Hospital, Ave. Doctor Clara 19, Castellon, 12002, Spain; 3 Oncology Service, Castellon Province Hospital, Ave. Doctor Clara 19, Castellon, 12002, Spain; 4 Plastic Surgery Service, Castellon Province Hospital, Ave. Doctor Clara 19, Castellon, 12002, Spain; 5 Nuclear Medicine Service, Castellon Province Hospital, Ave. Doctor Clara 19, Castellon, 12002, Spain; 6 Surgical Oncology Unit, Castellon Province Hospital, Ave. Doctor Clara 19, Castellon, 12002, Spain

## Abstract

**Background:**

Melanoma of the anal region is a very uncommon disease, accounting for only 0.2-0.3% of all melanoma cases. Mutations of the *BRAF *gene are usually absent in melanomas occurring in this region as well as in other sun-protected regions. The development of a tumour in a longstanding perianal fistula is also extremely rare. More frequent is the case of a tumour presenting as a fistula, that is, the fistula being a consequence of the cancerous process, although we have found only two cases of fistula-generating melanomas reported in the literature.

**Case Presentation:**

Here we report the case of a 38-year-old male who presented with a perianal fistula of four years of evolution. Histopathological examination of the fistulous tract confirmed the presence of malignant melanoma. Due to the small size and the central location of the melanoma inside the fistulous tract, we believe the melanoma reported here developed in the epithelium of the fistula once the latter was already formed. Resected sentinel lymph nodes were negative and the patient, after going through a wide local excision, remains disease-free nine years after diagnosis. DNA obtained from melanoma tissue was analysed by automated direct sequencing and the *V600E *(*T1799A*) mutation was detected in exon 15 of the *BRAF *gene.

**Conclusion:**

Since fistulae experience persistent inflammation, the fact that this melanoma harbours a *BRAF *mutation strengthens the view that oxidative stress caused by inflammatory processes plays an important role in the genesis of *BRAF *gene mutations.

## Background

Malignant melanomas of the anal region are a rare occurrence, accounting for only around 0.2-0.3% of all melanoma cases and 4% of anal cancers [1]. Diagnosis may be challenging, since perianal melanomas may be disguised as haemorrhoids [2] and more rarely as fissures [3] or perianal abscesses [4]. Prognosis is generally very poor [5], with five-year survival rates ranging between 6.7% and 16% [6,7].

The *BRAF *gene encodes a serine/threonine kinase involved in the mitogen-activated protein kinase pathway (MAPK) [8]. Activating *BRAF *mutations are present in approximately 70% of cutaneous malignant melanomas [9] and 82% of melanocytic naevi [10]. More than 90% of *BRAF *mutations involve a single point mutation, *T1799A*, in codon 600 of exon 15, leading to a *V600E *amino acid substitution [9,10]. However, several studies have shown that *BRAF *mutations are very uncommon in melanomas arising in sun-protected areas [11-13]. These findings have suggested an association between the presence of *BRAF *mutations in malignant melanomas and ultraviolet (UV) light exposure.

In this report we describe a case of malignant melanoma presenting in a perianal fistula. To the authors' knowledge, this clinical presentation has not been previously described in the scientific literature. To add to our knowledge of this rare entity, we have determined the mutational status of the *BRAF *gene in tumour tissue of the patient.

## Case presentation

**Figure 1:**
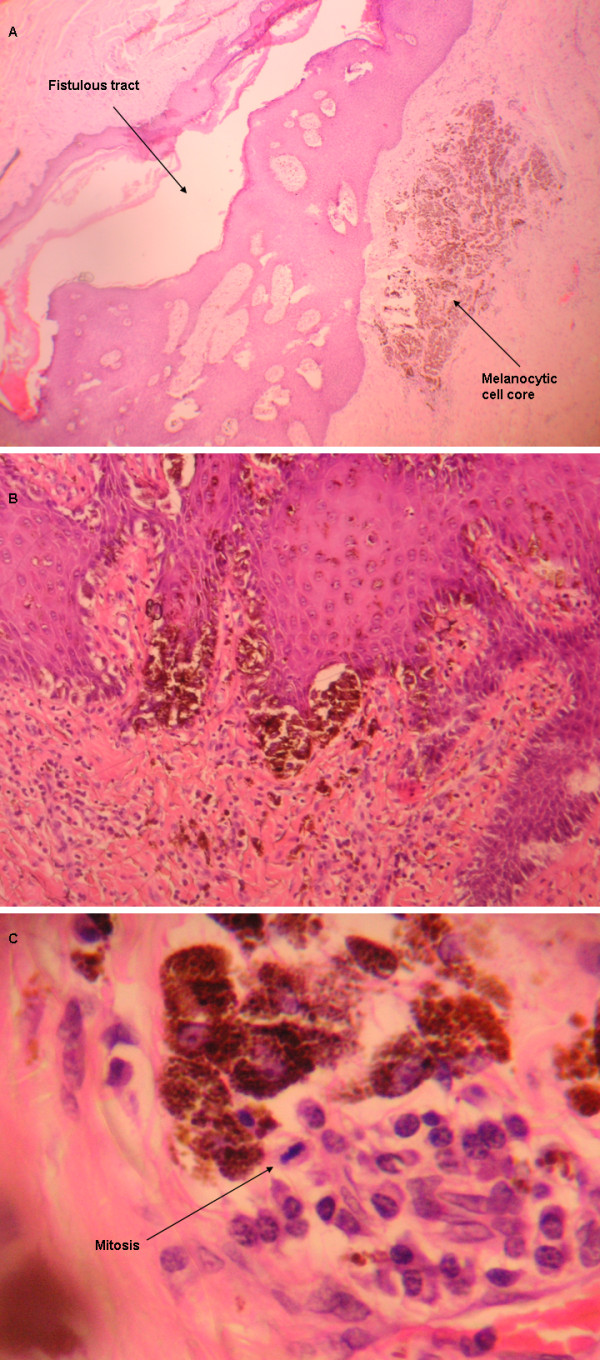
**Photomicrographic images of the fistula melanoma**. A) Fistulous tract course with nest of melanocytic cells (Haematoxylin-Eosin staining; magnification 4×). B) Closer view of the melanocytic cell core (H&E; magnification 10×). C) Melanoma cancerous cells, including one undergoing mitosis (H&E; magnification 40×).

The patient was a 38-year-old male with a history of beta-thalassaemia minor with pseudopolycythemia and microcytosis. In May 2002 he underwent surgery for a perianal fistula of four years of evolution. Pathologic examination of the resected specimen revealed an abscessed fistulous tract with the presence of a small core of hyperpigmented and hyperplastic basal melanocytes arranged irregularly and forming a lentiginous pattern (Figure 1). A nest of epithelioid cells with atypical anaplastic nuclei, prominent nucleoli and abundant melanin was clearly discernible in the underlying dermis (Figure 1). Besides, features relatively specific for melanoma including absence of maturation and presence of mitoses in deep cells were also visible (Figure 1). Finally, other secondary characteristic also suggestive of melanoma was the abundant melanic pigment throughout the lesion, both in the depth of the lesion and in the upper portion of the epidermis, including the stratum corneum or horny layer of the epidermis.

In June 2002, once the diagnosis of melanoma in perianal fistula (Clark level III) had been confirmed by the pathologist, the patient went through a surgical extension of excision margins (1 cm) and a sentinel lymph node biopsy (one right and two left inguinal nodes). Both the extended margins and the sentinel lymph nodes produced negative results for tumour cells.

In order to discard the possibility of metastatic melanoma, the patient was also subjected to an abdominal ultrasound examination and a whole body CT scan, both without relevant findings. Serum levels of S-100 protein, a widely used melanoma marker, were also negative. The patient went through his latest follow-up in December 2010, and at the time of this report he remains free of disease.

## Methods

### DNA extraction

Five μm sections from tumour tissue were stained with a standard Haematoxylin-Eosin protocol. Histological slides were examined with a Leica DM2000 microscope and micrographs were taken using a Leica DMD108 microimaging device with integrated camera (Leica Microsystems, Wetzlar, Germany). Slides were then cleaned with 99% ethanol for 15 minutes and left to air-dry overnight. Melanoma tissue was then manually dissected from surrounding normal tissue with a sterile blade. DNA extraction from dissected melanoma tissue was performed using the ChargeSwitch gDNA Tissue Kit (Invitrogen, Carlsbad, USA) according to the manufacturer's instructions.

### Detection of the *V600E *mutation

Melanoma DNA, a control sample (from peripheral blood of the same patient) and DNA extracted from melanoma cell line A375 were analysed for the *V600E *mutation at nucleotide 1799 of the *BRAF *gene by direct automated DNA sequencing. PCR primer sequences used to amplify a 102-bp fragment containing nucleotide 1799 of exon 15 were: 5'-GAAGACCTCACAGTAAAAATAGG TGA-3' (sense) and 5'-CCACAAAATGGATCCAGACA-3' (antisense). PCR reactions were carried out using about 100 ng of melanoma DNA as template. Thermocycling conditions included a denaturation step at 95°C for 8 minutes, followed by 40 cycles of 95°C for 30 seconds, 56°C for 45 seconds and 72°C for 1 minute, plus a final extension cycle of 72°C for 10 minutes. Amplified products were purified using ExoSAP-IT (USB Corporation, Cleveland, USA) according to the manufacturer's instructions. Purified PCR products were then run on an ABI 3130 Genetic Analyzer (Applied Biosystems, Foster City, USA) and analysed using software supplied by the manufacturer.

All new sequence data contained in this study has been deposited in the GenBank sequence database [GenBank:HQ224878].

Appropriate ethical approval for this research was obtained from the Ethics and Clinical Research Committee (CEIC) of the Castellon Province Hospital. Written informed consent was obtained from the patient for the study and its reportings.

## Results

**Figure 2:**
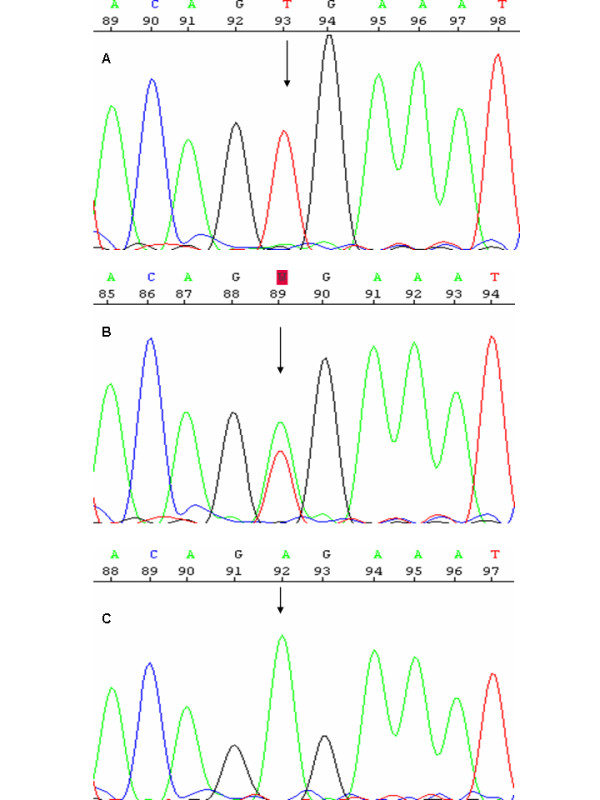
**Sequence chromatograms of the negative control, the fistula melanoma and the positive control DNA samples**. A) DNA from peripheral blood of the fistula melanoma patient with an arrow showing the wild-type genotype (the T allele in homozygosis). B) DNA from the perianal fistula melanoma showing the *T1799A *mutation in heterozygosis (arrow). C) DNA from melanoma cell line A375, with the mutation in the homozygous state.

High quality sequencing was obtained in both the 5' and the 3' direction from the melanoma sample, the control DNA sample and melanoma cell line A375. The *V600E *mutation (*T1799A*) in exon 15 of the *BRAF *gene was present in the heterozygous state in the melanoma sample, but it was absent in non-tumour DNA obtained from peripheral blood of the same melanoma patient (Figure 2). As expected, the positive control sample, melanoma cell line A375, showed the *V600E *mutation in the homozygous state (Figure 2).

## Discussion

The development of a tumour inside a longstanding fistula is an extremely infrequent event. More frequent is the case of a tumour presenting as a fistula, that is, the fistula being a consequence of the cancerous process. Occasionally, the inflammation and necrosis caused by the development and progression of a tumour stimulates the formation of a fistula. However, there are instances in which the fistula is not a secondary manifestation of the neoplasia, and its formation predates the development of the tumour. In these cases, the neoplastic transformation arises inside the longstanding fistula, probably due to the latter's chronic inflammatory nature [14]. We have found several cases in the literature in which a variety of different tumour types have developed inside a fistula once the latter was already formed, such as squamous cell carcinomas [15-17], basal cell carcinomas [18,19] and mucinous adenocarcinomas [17,20-22].

As far as the authors are aware, the occurrence of a melanoma within a fistula has not been previously described in the literature. We have found two published cases of melanomas presenting with fistulous formations, one concerning a biliary tract fistula [23] and another one presenting as a urethral fistula [24], in which the appearances of the fistulas were a consequence of the cancerous process.

**Figure 3:**
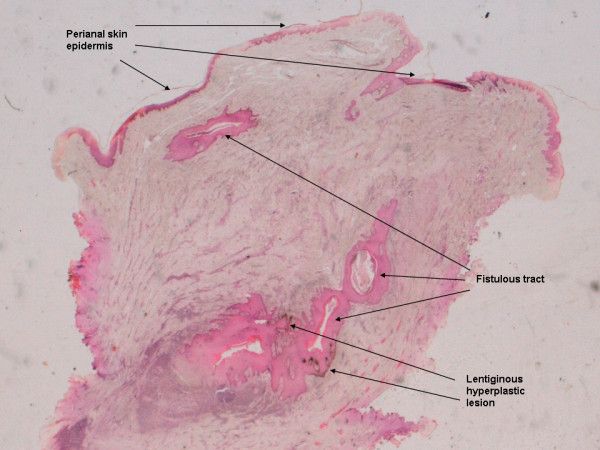
**Photomicrographic image of a cross-section of the entire resected fistula piece**. This picture of the entire resected piece (H&E; magnification 2×) shows the fistulous tract, the lentiginous melanocytic hyperplasia, and the perianal skin epidermis. Note that the lesion is located in the depth of fistula, not in the perianal epidermis.

In our patient, the fistula melanoma was quite small (Figure 1) and it was found by chance while the pathologist examined the stained histological sections of the fistula. The melanoma was located in the depth of the subcutaneous tissue, next to the fistulous tract, the interior of which was already epithelialised. It was surrounded by abundant inflammatory response and next to a lesion showing atypical lentiginous melanocytic hyperplasia (Figure 3). The presence of lentiginous hyperplasia suggested that the melanocytic lesion originated inside the fistulous tract and that we were indeed in front of a primary melanoma and not a metastasis (already ruled out by the whole body CT scan). On the other hand, the opening of the fistulous tract in the perianal skin epidermis did not contain signs of inflammation or malignant tissue (Figure 3), which ruled out the possibility of the fistula being a complication or a secondary manifestation of a cutaneous melanoma [14]. Therefore, we believe that the fistula predated the melanoma, not the other way around, and that it arose as a complication of the inflammatory lesion of the fistula.

Anal melanoma is an uncommon disease that affects patients of advanced age, usually appearing in the sixth or seventh decade of life [25,26]. However, Cagir and cols. found an increased incidence of anorectal melanomas in males aged 25 to 44 years living in the San Francisco area [27], and they proposed HIV infection as a risk factor for anorectal melanoma. But the HIV test in our patient (who was 38 years of age at diagnosis) was negative, and he had neither a previous history nor signs of other sexually-transmitted diseases.

The fact that this melanoma originated in a perianal location and inside a fistula also ruled out UV light exposure as a possible aetiological factor. Classic genetic mutations caused by UV exposure comprise C>T and CC>TT transitions, although UV exposure generates oxygen-free radicals that also damage DNA and give rise to other types of base substitutions [28]. Even though *BRAF *mutations occur more frequently in melanomas linked to sun exposure, the *BRAF *gene does not contain C>T or CC>TT transitions typical of UV-induced damage. The commonest *BRAF *mutation by far is *T1799A *(*V600E*), a T>A transversion present in more than 90% of all *BRAF*-mutated melanomas and naevi.

Due to the detection of the *V600E BRAF *mutation in this fistula melanoma, it was decided not to look for alterations in genes such as *CCND1 *or *KIT*, more frequently mutated in acral and mucosal melanomas [29], since concomitant mutations in *BRAF *and in genes such as *KIT *or *CCND1 *are extremely rare [29]. Regarding this, it has to be understood that, although this melanoma arose in the perianal region, it was not a case of mucosal melanoma, since it did not originate in the anal mucous membrane of the patient.

*BRAF *mutations, including *T1799A*, have occasionally been found in anal mucosal melanomas [30] and in other tumours not associated with UV exposure [9], such as thyroid cancer [31,32], colorectal cancer [33-35], pancreatic cancer [36,37] or ovarian cancer [38,39]. The development of all these tumour types may, in turn, be related to inflammatory processes [12], in the same way as cutaneous melanoma is associated with inflammation of the skin caused by UV exposure [40]. Accordingly, oxidative stress caused by inflammation may be in part responsible for mutations in the *BRAF *gene [12]. Since fistulae are environments that experience permanent inflammatory processes, the fact that the fistula melanoma reported here is carrying the *T1799A *transversion supports the hypothesis that oxidative damage caused by free radicals plays a role in the genesis of *BRAF *mutations.

Obviously, we are not presuming that all inflammation must cause *BRAF *mutations, but that persistent or chronic inflammatory processes in the absence of UV exposure (such as thyroiditis in thyroid cancer [31], chronic pancreatitis in pancreatic cancer [37], chronic bowel inflammation in colon cancer [41] and, in our case, a longstanding fistula in a melanoma), can probably make the *BRAF *gene more susceptible to genetic mutations via an as yet unidentified oxidative stress lesion.

As to the management of the perianal fistula melanoma, this case illustrates the benefits provided by the use of sentinel lymph node biopsy. Similarly to anorectal melanoma cases [42], the sentinel lymph nodes were located in the groin, not in the presacral region. As mentioned above, all three nodes dissected were free of tumour cells. After ruling out abdominoperineal resection [43], wide local excision (WLE) appears to obtain general good results when used as the initial treatment of choice in melanomas of the anal region [5,26]. Our case can only corroborate this, since nine years after WLE our patient remains healthy and without signs of relapse.

## Conclusions

The fact that the melanoma described in this report arose inside a fistula may set it apart from other anal and perianal melanomas, as well as from mucosal melanomas in general. In our opinion, and due to the symptoms occasioned by the presence of the fistula, this melanoma was caught at a very early stage compared to other anorectal or perianal melanomas. The presence of a *BRAF *mutation, usually absent in other anal melanomas, also suggests different aetiological factors contributing to the development of this particular melanoma, with inflammatory processes and oxidative stress damage being possibly among them.

## Competing interests

The authors declare that they have no competing interests.

## Authors' contributions

CM-C participated in the study design, performed the molecular genetic studies and sequence analysis, and drafted the manuscript. NB and LP performed the histopathological staining, took all photomicrographic images and participated in the draft of the manuscript. EF-C participated in the molecular genetic studies. EO participated in the study design and in manuscript critical revision. JM, JPA and MT participated in the study design and coordination, in the acquisition of clinical data and in manuscript revision. RR participated in the histopathological staining and in the study design. RL participated in the study design and coordination, as well as in manuscript revision. EB conceived the study, participated in its design and coordination and helped to draft the manuscript. All authors read and approved the final manuscript.

## Pre-publication history

The pre-publication history for this paper can be accessed here:

http://www.biomedcentral.com/1471-2407/11/343/prepub
